# Supplementary figures and images for: The global burden of Chikungunya fever among children: A systematic literature review and meta-analysis
Source: PLOS Glob Public Health. 2022 Dec 21;2(12):e0000914. doi: 10.1371/journal.pgph.0000914 (PMC10022366; doi:10.1371/journal.pgph.0000914)

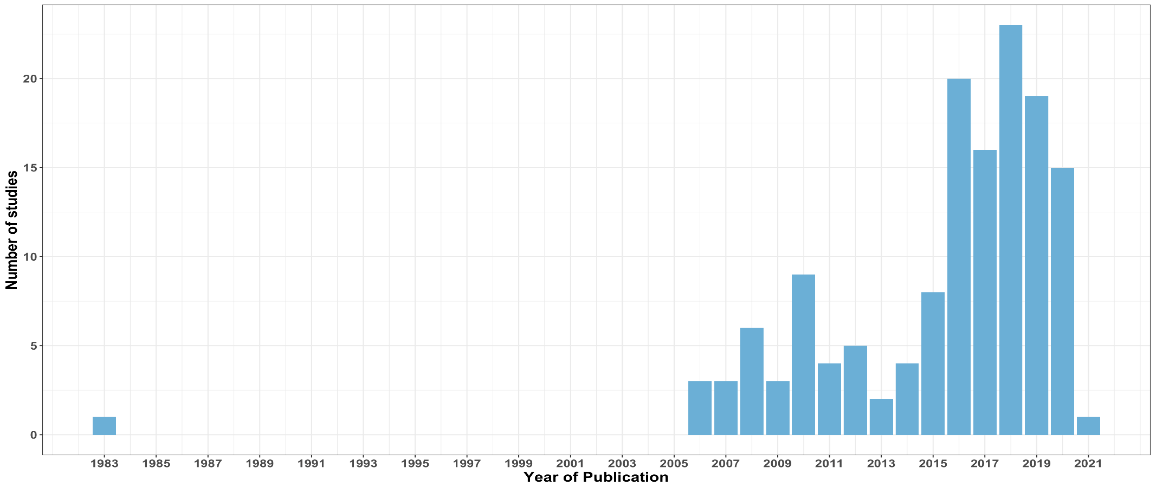

Supplement: S1 Fig — (TIF) [file pgph.0000914.s002.tif]
