# Supplementary material for: The global burden of Chikungunya fever among children: A systematic literature review and meta-analysis
Source: PLOS Glob Public Health. 2022 Dec 21;2(12):e0000914. doi: 10.1371/journal.pgph.0000914 (PMC10022366; doi:10.1371/journal.pgph.0000914)
Supplement: S5 Table — (DOCX) [file pgph.0000914.s007.docx]

| **Supplementary Table 6: Characteristics of the best five models fitted by the multimodel inference in metaregeression** | | | | | | | | | | | | | |
| --- | --- | --- | --- | --- | --- | --- | --- | --- | --- | --- | --- | --- | --- |
| **Model** | **Intc** | **Continent** | **Diagnostic Technique** | **Study design** | **Season** | **Sample size** | **Study duration** | **Year** | **DF** | **Loglik** | **AICc** | **Delta** | **Weight** |
|  |  |  |  |  | **(Epidemic)** |  |  |  |  |  |  |  |  |
| **256** | _+_ | _+_ | _+_ | _+_ | _+_ | 2.53E-15 | 1.86E-13 | -1.47E-12 | 34 | -5279.83 | 10628.6 | 0 | 0.299 |
| **128** | + | _+_ | _+_ | _+_ | _+_ | 6.22E-19 | -4.02E-16 |  | 33 | -5281.3 | 10629.4 | 0.88 | 0.192 |
| **248** | + | _+_ | _+_ | _+_ | _+_ |  | -3.52E-13 | 1.33E-09 | 33 | -5281.3 | 10629.4 | 0.88 | 0.192 |
| **224** | _+_ | _+_ | _+_ | _+_ | _+_ | -2.70E-16 |  | 9.42E-13 | 33 | -5281.3 | 10629.4 | 0.88 | 0.192 |
| **216** | _+_ | _+_ | _+_ | _+_ | _+_ |  |  | -4.04E-13 | 32 | -5282.76 | 10630.3 | 1.76 | 0.124 |
| [*A number (weight) or + sign (for categorical predictors) indicates that a predictor/interaction term was used in the model, while empty cells indicate that the predictor was omitted](https://rdrr.io/r/base/Arithmetic.html) | | | | | | | | | | | | | |
