# Supplementary material for: The global burden of Chikungunya fever among children: A systematic literature review and meta-analysis
Source: PLOS Glob Public Health. 2022 Dec 21;2(12):e0000914. doi: 10.1371/journal.pgph.0000914 (PMC10022366; doi:10.1371/journal.pgph.0000914)
Supplement: Attachment — Submitted filename: Plos_GH_Author_Response_corrected_DKN.docx [file pgph.0000914.s008.docx]

Doris Nyamwaya

Research Officer

CGMRC, PO Box 230 – 80108,

Kilifi, Kenya

Phone: +254 737099550

Editorial Office

*PLOS Global Public Health*

**22 July 2022**

**Ref: PGPH-D-22-00382 (The global burden of Chikungunya fever among children: A systematic literature review and meta-analysis)**

Thank you for reviewing our manuscript. We are very pleased for inviting us to submit a revised version of the manuscript that addresses the points raised during the review process.

We have addressed all the comments that have been raised in the decision letter. Our specific responses to each item of feedback are listed point-by-point in page two of this document (highlighted in yellow).

Please let us know if any further information or discussion is needed, otherwise we will look forward to hearing back from you in due course.

Yours sincerely,

**Doris Nyamwaya (corresponding author)**

KEMRI-Wellcome Trust Research programme,

Kilifi, Kenya

**Ref: JVH-00065-2021**

**Editor comment one**

Figure 2: please (a) provide a direct link to the base layer of the map used and ensure this is also included in the figure legend; (b) provide a link to the terms of use / license information for the base layer. We cannot publish proprietary or copyrighted maps (e.g. Google Maps, Mapquest) and the terms of use for your map base layer must be compatible with our CC-BY 4.0 license.

If your map was obtained from a copyrighted source please amend the figure so that the base map used is from an openly available source. Alternatively, please provide explicit written permission from the copyright holder granting you the right to publish the material under our CC-BY 4.0 license.

Please note that the following CC BY licenses are compatible with PLOS license: CC BY 4.0, CC BY 2.0 and CC BY 3.0, meanwhile such licenses as CC BY-ND 3.0 and others are not compatible due to additional restrictions.

If you are unsure whether you can use a map or not, please do reach out and we will be able to help you. The following websites are good examples of where you can source open access or public domain maps: 
* U.S. Geological Survey (USGS) - All maps are in the public domain. (<http://www.usgs.gov>) 
* PlaniGlobe - All maps are published under a Creative Commons license so please cite “PlaniGlobe, <http://www.planiglobe.com>, CC BY 2.0” in the image credit after the caption. (<http://www.planiglobe.com/?lang=enl>) 
* Natural Earth - All maps are public domain. (<http://www.naturalearthdata.com/about/terms-of-use/>)

Our response is as follows:

Figure 2 was generated using a freely available R software. We have provided a link to the base layer of the map and included this in the legend as “The map was generated using open-source data in the R statistical package “spData” (https://cran.r-project.org/web/packages/spData/index.html)”

**Editor comment two**

Thank you for addressing Chikungunya. This is a very important topic and one I care very much about.

Thank you for your positive feedback

I would reclassify locations as having a) novel introductions b) epidemics c) endemic d) mix e) none.

The first category has been an important one in ChikV dynamics over the last 15 or so years. As ChikV traveled with its new mosquito friends (Aedes albopictus) to Reunion and Kerala and elsewhere in India, and then on to the Caribbean and Central America and on to Brazil and the rest of South America, we saw dramatic case numbers. Whole communities had ChikV all at once. There are areas where no one living there did not have the "fever". The high viremia with ChikV means it has a very rapid "urban" transmission cycle with a very high rate of symptomatics. After a wave, the "fever" dies out because there are not enough susceptibles given the high attack rate and immunity is long lasting from a single infection (though of course we may see different strains behave differently). If transmission persists, there may be new, but smaller, surges as susceptibles build up and there are enough small children, new migrants, and perhaps some protected persons (away during first surge or in an AC- house).

Our response is as follows:

The studies we included in this review were either carried out during an epidemic or non-epidemic season while some of the studies did not specify the season. However, most descriptions of an epidemic included novel introductions into naïve populations. We agree that this forms an important factor in the synthesis of CHIKV transmission dynamics. We have summarised this in lines 313-319 as follows:

“Spread of CHIKV into naïve populations in the Indian ocean islands, south east Asia and then to the Americas and the carribbean islands, resulted into rapid establishment of local transmission and high clinical attack rates among children [1–3]. The intensity of transmission has been associated with disease severity[4]. The CHIKV force of infection and associated clinical manifestations are influenced by the exposure dose. [1,3]. The epidemics resulted into significant absenteesm among school-going children, excessive demand for health care, marked economic losses and significant morbidity and mortality [1]. Neonates were disproportionately affected [1]”

**Editor comment three**

I would not highlight the 11% figure. This will likely be misread and could lead to a misunderstanding about ChikV dynamics. Given all the work that's gone into this paper, I would not want that to happen.
Instead as ChikV crashes as a surging wave in areas without prior immunity, averages, just like we have seen with COVID surges, would not be as meaningful. Instead, cumulative prevalence would be interesting, but that's not available. I would instead highlight what endemic rates are, which is important and quite high, and also point to how high these can be in novel outbreaks. I don't think pooling these would help explain this to readers though.

This is much like trying to capture COVID surges and the difficulty of point prevalences. We saw how devastating COVID can be in a population with no immunity and with increase travel and climate/environment change we will presumably see more so this is an important topic being discussed here beyond just ChikV. I would think of this as like when Omicron first caused sudden large surges; it may not be the norm after. Endemic areas the rate would be more meaningful as it has reached more of a steady state, rather than trying to capture a rate of a moving target in a surge.

We have included this table to show the rates of CHIKV cases in each continent in lines 207-209.

Table 3: The rates of CHIKV cases reported among children from various continents in different seasons

| Continent | Season n/N (%) CHIKV Cases | | | |
| --- | --- | --- | --- | --- |
|  | Both | Epidemic | Non-epidemic | unspecified |
| Africa | 32/383  (8.36) | 516/4729  (10.91) | 124/2011  (6.17) | 43/749  (5.74) |
| Asia | - | 425/1954  (21.75) | 31/1915  (1.62) | 1087/14534  (7.48) |
| C. America | - | 96/3377  (2.84) | - | - |
| Carribean | - | 154/275  (56) | 74/1390  (5.32) | - |
| Indian ocean islands | - | 224/2959  (7.57) | - | - |
| Oceania | - | 13/33  (39.39) | - | - |
| S. America | - | 83/205  (40.49) | - | 194/641  (30.27) |

**Editor comment four**

I would also try to bring in some of the information these papers bring about the specifics of the cases in pediatrics. There was a lot of rich information on case presentations in the literature cited here, especially regarding infants and congenital cases. On page 9 and 10 many factors are listed but more discussion would help (especially as some factors are in opposition to some degree like neutrophila and leukopenia). It is helpful that thrombocytopenia is stressed and often is not recognized.

We have provided further explanation of the clinical presentation in lines 248-256

“The clinical presentation of paediatric CHIKF varies with geographical location and the circulating strain of CHIKV. In Reunion island where epidemics were predominantly due to the Indian ocean lineage, majority of infected neonates had thrombocytopenia , deranged coagulation, seizures , hemodynamic disorders and hemorrhagic syndromes [9]. In contrast, circulatory failure was common among CHIKV infected infants in Kerala where the ECSA strain circulated [3]. The Asia genotype introduced to the Carribbean islands and central America induced weaker pro-inflammatory effect with a milder clinical course among infected neonates. It was not associated with thrombocytopaenia and clinical neurological sequale, however the case fatality rate was comparably high [10,11] “

**Editor comment five**

I would also consider reformatting how geographic distributions are discussed. Many of the cases in Europe and North America (outside of the Caribbean and Central American countries) were from travel and I would (though it is mentioned for Europe) carve this would a separate category. Moreover, there are also many in the Caribbean and Central America who would not describe themselves as in North America. Given the epidemiological dynamics of ChikV were so different in Central America and the Caribbean, I might make Central America and Caribbean a separate category (or categories). It would be better understood by people in these areas and also reflect the climate differences that are quite important here.

I might discuss more about the reasons for geographic differences (mosquito change and climate).

We have reformatted the discussion on geographical distribution of cases.

Majority of the cases in Europe and North America were associated with travel-importation of the infection. Studies from the Carribbean islands and Central America focused on describing epidemiological and clinical aspects of epidemic waves following introduction of the virus into naïve population (see lines 181 -185). We have also noted the geographical differences in clinical manifestations that are attributed to the circulating CHIKV genotype in lines 348 - 355.

**Reviewer one: comment one**

The study was carried out on “The global burden of Chikungunya 1 fever among children: A systematic literature review and meta-analysis” and provided a detailed information on Chikungunya and clinical facts in <18 years age group. The study was done systematically and data analyzed properly. The major comments are as follows:

• There is lot of literature is available on Chikungunya fever illness, however authors has not much focused on epidemiology, geographical distribution and pathophysiology of diseases in introduction part.

We have revised the introduction and we feel we have covered this area sufficiently.

**Reviewer one: comment two**

Why only <18 age group. What difference was observed from published literature between <18 and >18 groups in Chikungunya clinical conditions.
• Authors should have mentioned a paragraph on differences between <18 and >18 chikungunya clinical as well as treatment conditions.

The focus of this review was on paediatric CHIKF, as outlined in lines 107-110.

“Nevertheless, descriptions on the clinical spectrum and epidemiology of paediatric CHIKF, particularly in Africa are limited. We therefore set out to describe the epidemiology of CHIKF among children (aged <18 years) globally by describing the proportion of acutely unwell children who tested positive for CHIKV, geographical distribution, and clinical manifestations through a systematic literature review and meta-analysis.”

**Reviewer one: comment three**

Clinical condition between different geographic conditions should be included which might have provide a good knowledge (genetically) further to understand the molecular epidemiology of Chikungunya.

Differences between paediatric CHIKV and adult CHIKF are highlighted in lines 93-101

“There are few studies that have described the clinical manifestations of paediatric CHIKF, which tend to differ from manifestations described in adults [20,22–24]. After an incubation period ranging 1 to 12 days, there is a sudden onset of high-grade fever. In adults, this is usually preceded by musculoskeletal manifestations including myalgia, back pain and symmetric arthropathy affecting distal joints that can be sometimes chronic [20]. In contrast, children exhibit a broader range of cutaneous manifestations like pigmentation, bullous rash and blistering [25]. Neurological manifestations including seizures, encephalopathies and meningoencephalitis are common among children. Haemorrhagic manifestations associated with thrombocytopenia and lymphopenia are commonly reported in paediatric CHIKF [26].”

**Reviewer two: comment one**

This is a well-written review and meta-analysis that underscores the importance of improving surveillance for this relatively neglected viral disease.

Thank you for your positive feedback

My suggestions are minor:
1. Abstract / Findings / line 50-52 -- there are a couple of confusing elements in here and I suggest rewriting to clarify. E.g., the statement "the pooled positivity rate among acutely unwell children was 11%" implies that this was the rate among all unwell children, and the text and tables suggest that the highest rate was from South America (22.8%), not North America (22. 2%), but in any case, these two estimates are so close that perhaps they should be combined to "the Americas".

We have removed the description of pooled positivity rates from the manuscript to avoid misunderstanding of CHIKV dynamics. This was in response to the important suggestion by the editor

**Reviewer two: comment two**

49: change 'least' to 'fewest'; add 'more' -- "included studies were more commonly conducted"
64: positive-sense
66: change 'could' to 'may'
67: delete colon
74: delete 'within', add 'the' -- "the Pacific Islands"
81: add comma after 'underestimated'
88: subject-verb agreement 'there remains limited descriptions'
91: tested positive 'for CHIKV'
100: capitalize Chikungunya
108: add parentheses (DKN and JM)
109: laboratory-confirmed
110: replace 'those' with 'and'
116: Google Sheets
118-119: what if a study spanned both epidemic and non-epidemic seasons?
131: delete 'and' -- "population that screened participants"
136: superscript the 2 in r2
137: intervals (with an 's')
148: significant
154: delete 'being'
203: leukopenia (to be consistent with spelling elsewhere)
211: please write out numbers at the start of a sentence -- "Sixty-five of one hundred forty-two studies"
231: I believe the English term for 'apalastesis' is pallesthesia
242-244: confusing -- there are 16 included studies from the Americas, so why do the authors refer to one study from the Americas after 2013? I fear I am missing their point.
243: 'relatively' few studies -- because by number, there are quite a few, but it may be that there are few relative to the number of CHIKF cases in these regions
255: replace 'indicates' with 'suggests'?
260: born to viremic mothers
261: CHIKV-infected
263: set-ups, as
269: call
277: delete 'the'
Figure 3: captions refer to "CHIKV cases" which is a bit confusing as I thought the disease was CHIKF and the virus was CHIKV -- please clarify what's meant here by CHIKV cases

We have considered all the proofreading suggestions as indicated
