# Supplementary material for: The global burden of Chikungunya fever among children: A systematic literature review and meta-analysis
Source: PLOS Glob Public Health. 2022 Dec 21;2(12):e0000914. doi: 10.1371/journal.pgph.0000914 (PMC10022366; doi:10.1371/journal.pgph.0000914)
Supplement: Attachment — Submitted filename: Rebuttal_ letter2_Plos_GH_DKN_12102022.docx [file pgph.0000914.s009.docx]

Doris Nyamwaya

Research Officer

CGMRC, PO Box 230 – 80108,

Kilifi, Kenya

Phone: +254 737099550

Editorial Office

*PLOS Global Public Health*

**12 September 2022**

**Ref: PGPH-D-22-00382 (The global burden of Chikungunya fever among children: A systematic literature review and meta-analysis)**

Thank you for reviewing our manuscript. We are very pleased for inviting us to submit a revised version of the manuscript that addresses the points raised during the second review process.

We have addressed all the comments that have been raised in the decision letter. Our specific responses to each item of feedback are listed point-by-point in page two of this document (highlighted in yellow).

Please let us know if any further information or discussion is needed, otherwise we will look forward to hearing back from you in due course.

Yours sincerely,

**Doris Nyamwaya (corresponding author)**

KEMRI-Wellcome Trust Research programme,

Kilifi, Kenya

**Editor comment one**

Journal Requirements:
Please review your reference list to ensure that it is complete and correct. If you have cited papers that have been retracted, please include the rationale for doing so in the manuscript text, or remove these references and replace them with relevant current references. Any changes to the reference list should be mentioned in the rebuttal letter that accompanies your revised manuscript. If you need to cite a retracted article, indicate the article’s retracted status in the References list and include a citation and full reference for the retraction notice.

We have reviewed the reference list and its complete and correct. We have also checked using the Scite’s reference check tool (<https://scite.ai/)> that indicated there are no references that have been retracted nor with editorial concern.

**Editor comment two**

antibiotics/bacteria
You mention in the abstract "antibiotics for presumed secondary bacterial infection", but there is not much more about antibiotics and secondary bacterial infections in the paper. It is mentioned "234 prescription was predominantly given to children presenting with undifferentiated fever before CHIKF 235 was diagnosed as reported in 35/65 studies" and it is show in the but this is different than treating bacterial superinfections, this is empiric treatment and misdiagnosis. Is there any information on secondary bacterial infections? In my experience, these are rather rare. I might adjust the abstract to match the paper more closely.

We have adjusted the abstract as follows; (lines 52-54)

“Children with undifferentiated fever before CHIKF was diagnosed were treated with antibiotics ~~for presumed secondary bacterial infection,~~ and/or drugs that manage specific symptoms or those that provided supportive care.”

**Editor comment three**

2. genotypes and different chikv types
You may want to include a table (or map) showing the differences suspected between genotypes and where they have spread and which mosquitoes involved.

This is a great suggestion. This map was generated and published here [https://www.nejm.org/doi/full/10.1056/NEJMra1406035](https://eur01.safelinks.protection.outlook.com/?url=https%3A%2F%2Fwww.nejm.org%2Fdoi%2Ffull%2F10.1056%2FNEJMra1406035&data=05%7C01%7CDNyamwaya%40kemri-wellcome.org%7Cfc707fd107554e9ddce108daabb71c23%7Ca5c0a820c8874727ac66403237d8c389%7C0%7C0%7C638011100187533409%7CUnknown%7CTWFpbGZsb3d8eyJWIjoiMC4wLjAwMDAiLCJQIjoiV2luMzIiLCJBTiI6Ik1haWwiLCJXVCI6Mn0%3D%7C3000%7C%7C%7C&sdata=c3DyhXTvM30l8xK5zXQEcehU%2BqWXc4lkQk%2BPQDjZb9s%3D&reserved=0) (Figure 2). We can seek approvals from NEJM to reuse the figure if need be. The kind of data we collected in this review does not provide for generating our map/table with this information.

You state in the intro "There are 64 three main CHIKV genotypes, the west African (WA), the east central and southern African (ECSA) 65 and the Asian genotypes [4]. Mutations in the ECSA genotype resulted in the Indian ocean lineage 66 (IOL) [5]." I would add more in the intro to flesh this out.
I would avoid saying "It is commonly caused by the ECSA and IOL 294 genotypes" as it makes it seem like ECSA and IOL are different. I might try to explain more if ECSA is thought to be the cause or whether this was seen more in the Indian Ocean but more just try to explain more in the intro about the different genotypes and their associations.

We have included a description of the genotypes in the intro (lines 67-72)

“There are three main CHIKV genotypes, the west African (WA) , the east central and southern African (ECSA) and the Asian genotypes [10]. The strains of the WA lineage are maintained in a sylvatic cycle in western Africa countries with small focal spillover outbreaks of human infections reported [11,12]. The ECSA strains are enzootic in East, Central and Southern Africa but they have been detected in samples from a broader geographical range [13]. Mutations in the ECSA genotype resulted in the Indian ocean lineage (IOL) [13] .”

We have replaced this phrase "It is commonly caused by the ECSA and IOL” as; line 302

“It was observed more among children infected by the ECSA and IOL genotypes”

I would add some info on the differences in genotypes outside of pediatrics to intro and leave differences in pediatrics for the end.

We have included a description of the genotypes in the intro (lines 65-70)

“There are three main CHIKV genotypes, the west African (WA) , the east central and southern African (ECSA) and the Asian genotypes [10]. The strains of the WA lineage are maintained in a sylvatic cycle in western Africa countries with small focal spill-over outbreaks of human infections reported [11,12]. The ECSA strains are enzootic in East, Central and Southern Africa but they have been detected in samples from a broader geographical range [13]. Mutations in the ECSA genotype resulted in the Indian ocean lineage (IOL) [13] .”

I might clarify what milder meant (esp as health surveillance data varies), but could add this sort of info to the intro, except for the thrombocytopenia in children.
Studies in the Carribbean Islands and Central America where outbreaks were caused by the
291 Asian genotype, indicated a milder course of disease [47,48] when compared to those from Reunion 292 islands where the Indian Ocean lineage circulated [85,98]. CHIKF associated thrombocytopenia in 293 children dependent on the infecting CHIKV genotype.

We have deleted this sentence (299 – 301): “Studies in the Carribbean Islands and Central America where outbreaks were caused by the Asian genotype, indicated a milder course of disease [47,48] when compared to those from Reunion islands where the Indian Ocean lineage circulated [85,98]” to focus in the clinical differences observed in the paediatric population.

I would also clarify more about the distribution, as ECSA was introduced into the Americas. <https://pubmed.ncbi.nlm.nih.gov/33861753/>

We have included/revised the description of the genotypes in the intro (lines 65-79)

“There are three main CHIKV genotypes, the west African (WA) , the east central and southern African (ECSA) and the Asian genotypes [10]. The strains of the WA lineage are maintained in a sylvatic cycle in western Africa countries with small focal spillover outbreaks of human infections reported [11,12]. The ECSA strains are enzootic in East, Central and Southern Africa but they have been detected in samples from a broader geographical range [13]. Mutations in the ECSA genotype resulted in the Indian ocean lineage (IOL) [13] .

The first CHIKF outbreak was reported in Tanzania (1952) and was followed by several sporadic and major epidemics in tropical and subtropical regions; Africa, Asia, Europe, the Pacific islands and the Americas [25]. Epidemics were mostly experienced in 1960s and 1990s but the 2004 CHIKV epidemic (caused by genotype ECSA), emerged in coastal Kenya and is the largest on record reporting over 1.3 million cases [11,26]. It spread along the Kenyan coast from Lamu to Mombasa and islands on the Indian Ocean [27,28], India and subsequently to Southeast Asia and Europe [11,16,29]. This rapid expansion into new ecological niches follows adaptation of CHIKV to new mosquito vectors and their introduction into temperate regions [14,30] as well as travel-associated importation of the virus into naïve populations [16] resulting in outbreaks associated with high morbidity and mortality [31]” ‬‬

**Editor comment Four**
The systematic review does in figure 1 explain why studies were included/excluded. You can provide more clarifications as requested by Reviewer 2.

We have clarified in lines 170-173 as;

“Our search identified 2104 unique articles. After screening the titles, abstracts and full texts, 142 articles met the inclusion criteria; following exclusion of articles that; did not focus on children, did not describe acute CHIKV infections, were inaccessible, reviews, conference abstracts and general outbreak descriptions (Fig 1). ”

**Editor comment five**
Do try to, as Reviewer 1 states, to "add information about the epidemiology, geographical distribution and pathophysiology of disease"

We have added information on

**Epidemiology and geographical distribution (lines 65 -79)**

“There are three main CHIKV genotypes, the west African (WA) , the east central and southern African (ECSA) and the Asian genotypes [10]. The strains of the WA lineage are maintained in a sylvatic cycle in western Africa countries with small focal spillover outbreaks of human infections reported [11,12]. The ECSA strains are enzootic in East, Central and Southern Africa but they have been detected in samples from a broader geographical range [13]. Mutations in the ECSA genotype resulted in the Indian ocean lineage (IOL) [13] .

The first CHIKF outbreak was reported in Tanzania (1952) and was followed by several sporadic and major epidemics in tropical and subtropical regions; Africa, Asia, Europe, the Pacific islands and the Americas [25]. Epidemics were mostly experienced in 1960s and 1990s but the 2004 CHIKV epidemic (caused by genotype ECSA), emerged in coastal Kenya and is the largest on record reporting over 1.3 million cases [11,26]. It spread along the Kenyan coast from Lamu to Mombasa and islands on the Indian Ocean [27,28], India and subsequently to Southeast Asia and Europe [11,16,29]. This rapid expansion into new ecological niches follows adaptation of CHIKV to new mosquito vectors and their introduction into temperate regions [14,30] as well as travel-associated importation of the virus into naïve populations [16] resulting in outbreaks associated with high morbidity and mortality [31]. ‬‬

**Pathophysiology (lines 80-92)**

*“*CHIKV is inoculated by an infected mosquito bite either on human resident dermal cells i.e fibroblasts and macrophages, or directly unto the blood circulatory system [4,5]. Initial replication takes place in these skin cells triggering an immune response. It then disseminates to draining lymph nodes for further replication, and spread to other peripheral organs including muscle, peripheral joints, and tendons [6]. In severe cases, the virus invades the brain and liver [6,7].

CHIKF is characterised by a sudden rise in body temperature and debilitating joint pain that may resolve within weeks or persist for months to years [17]. Other symptoms include; headache, maculo-papular rash, fatigue, myalgia, backache and tachycardia [11,18]. Severe complications like encephalitis, myocarditis, kidney dysfunction, hepatitis, oculitis, cardiovascular and respiratory disorders have been observed, and are more common among the elderly, infants and immunosuppressed individuals [17,19]. There are currently no specific antivirals or approved vaccines for CHIKV infection, though efforts towards vaccine development are underway [20–24].”

**Reviewer one: comment one**

Page 12, line 274: Correct the spelling of ‘mortality’

This is corrected

**Reviewer one: comment two**

The added information about the epidemiology, geographical distribution and pathophysiology of disease is not sufficient. Must focus on recent literature about global epidemiology and geographical distribution of chikungunya fever.

We have added information as indicated on the response to editor comment five above

**Reviewer one: comment three**

The authors should include a paragraph highlighting various clinical conditions among different geographic conditions.

This information is described in lines 226-234 as;

“The clinical presentation of paediatric CHIKF varies with geographical location and the circulating strain of CHIKV. In La Reunion island where epidemics were predominantly due to the IOL, majority of infected neonates had thrombocytopenia , deranged coagulation, seizures , hemodynamic disorders and hemorrhagic syndromes [98]. In contrast, circulatory failure was common among CHIKV infected infants in Kerala where the ECSA strain circulated [99]. The Asia genotype introduced to the Carribbean islands and central America induced weaker pro-inflammatory effect with a milder clinical course among infected neonates. It was not associated with thrombocytopaenia and clinical neurological sequelae, however the case fatality rate was comparably high [100,101]”

**Reviewer three: comment one**

The manuscript presented may be of interest, however, I have some major issues with how the study was conducted.
1. The statement in the Background section states, "...we conducted a systematic literature review and meta-analysis to determine the epidemiology of CHIKF in children globally...".
I have an issue here since in many parts of the world there are socio-demographic conditions that can heavily impact transmission and even the severity of the disease (access to good health care).

Thank you for your feedback, by globally we mean that we included studies from all over the world and described the epidemiology of CHIKF among children (aged <18 years) based on these studies.

We acknowledge that there would be differences/biases based on socio-demographic conditions of different settings which can impact on transmission dynamics and disease severity. We have included this as a limitation in line 321-323.

**Reviewer three: comment two**

In the Findings section, it states that of the 2104 studies only 142 and 53 were included in the systematic literature review and meta-analysis. It is not clear why.

We have revised our abstract to state: “Our search identified 2104 studies, of which 142 and 53 articles which met the inclusion criteria were included in the systematic literature review and meta-analysis, respectively.” (lines 47-48)

We have further expounded this in the main text lines 170 – 173

“Our search identified 2104 unique articles. After screening the titles, abstracts and full texts, 142 articles met the inclusion criteria; following exclusion of articles that; did not focus on children, did not describe acute CHIKV infections, were inaccessible, reviews, conference abstracts and general outbreak descriptions”

In the conclusions, it says that it is an underreported health problem among children. This is actually true for most (if not all) infectious diseases since young children have a developing immune system.

Thank you for this comment, which we have mentioned in our discussion line 288

“Neonates were disproportionately affected owing to their developing immune system”
